# Priming the Brain Before Chemotherapy: Chronic Stress and HPA-Axis Dysregulation in Cancer-Related Cognitive Impairment

**DOI:** 10.3390/brainsci16101071

**Published:** 2026-10-06

**Authors:** Lijo Andrews, Yasmin El Khaldi, Tracy George, Tala Abuhamdah, Nebojsa Nick Knezevic

**Affiliations:** 1 Department of Anesthesiology, Advocate Illinois Masonic Medical Center, Chicago, IL 60657, USA; lijo.andrews@midwestern.edu (L.A.); yasmin.el.khaldi@gmail.com (Y.E.K.); tracy.george@midwestern.edu (T.G.); tala.abuhamda1@gmail.com (T.A.); 2 Chicago College of Osteopathic Medicine, Midwestern University, Downers Grove, IL 60515, USA; 3 Department of Anesthesiology, University of Illinois, Chicago, IL 60612, USA; 4 Department of Surgery, University of Illinois, Chicago, IL 60612, USA

**Keywords:** cancer-related cognitive impairment, hypothalamic–pituitary–adrenal (HPA) axis, neuroinflammation, chronic stress, cortisol, glucocorticoids, chemobrain

## Abstract

Cancer-related cognitive impairment (CRCI) is often attributed to chemotherapy, yet cognitive deficits are detectable in some patients before systemic treatment begins. This review examines a proposed two-hit framework in which cancer-related psychological and physiological stress contributes to pretreatment neural vulnerability, followed by superimposed chemotherapy-associated neurotoxicity. The review focuses on adults with non-central nervous system cancers. This narrative review synthesizes epidemiologic, neuroendocrine, neuroimaging, neuroimmune, autonomic, preclinical, and clinical evidence addressing chronic stress biology, pretreatment cognitive and brain changes, chemotherapy-related neurotoxicity, hypothalamic–pituitary–adrenal (HPA) axis interactions, and potential interventions. Pretreatment cognitive impairment has been reported in approximately one-quarter of patients, accompanied by elevated inflammatory cytokines, altered neurotrophic signaling, and changes in amygdala–hippocampal connectivity. The first hit may reflect interacting cancer- and host-related factors, including systemic inflammation, distress, sleep disruption, fatigue, pain, and HPA-axis/circadian dysregulation. The second hit occurs when chemotherapy inflicts superimposed neurotoxic injury, including white matter degradation, suppressed hippocampal neurogenesis, and blood–brain barrier disruption. The relationship may also be bidirectional: chemotherapy can perturb HPA-axis function, while glucocorticoid signaling may influence treatment response and antitumor immunity. Behavioral, autonomic, and circadian interventions are mechanistically promising, although neuroprotective efficacy remains uncertain. CRCI is likely multifactorial and may begin before chemotherapy. The two-hit framework integrates pretreatment stress-related vulnerability with treatment-associated neurotoxicity but remains hypothesis-generating rather than causally established. Prospective studies beginning at diagnosis and incorporating serial cognitive, psychological, endocrine, inflammatory, autonomic, and neuroimaging measures are needed to test this model and identify patients at greatest risk.

## 1. Introduction

Cancer-related cognitive impairment (CRCI) is often framed primarily as a consequence of chemotherapy, as reflected in the popular term “chemobrain.” However, cognitive difficulties can be present before systemic treatment begins, indicating that chemotherapy is not required for cognitive change and that later symptoms cannot automatically be attributed to treatment toxicity alone [1,2]. Pretreatment cognitive vulnerability is likely multifactorial, involving cancer-related biological processes together with psychological distress, fatigue, sleep disruption, pain, comorbid conditions, and other clinical factors [2,3].

Chemotherapy may then add a second exposure. Post-treatment studies have documented cognitive changes and alterations in brain structure and functional connectivity, although findings vary according to cancer type, treatment regimen, timing of assessment, and the methods used to define impairment [4,5].

**Figure 1:**
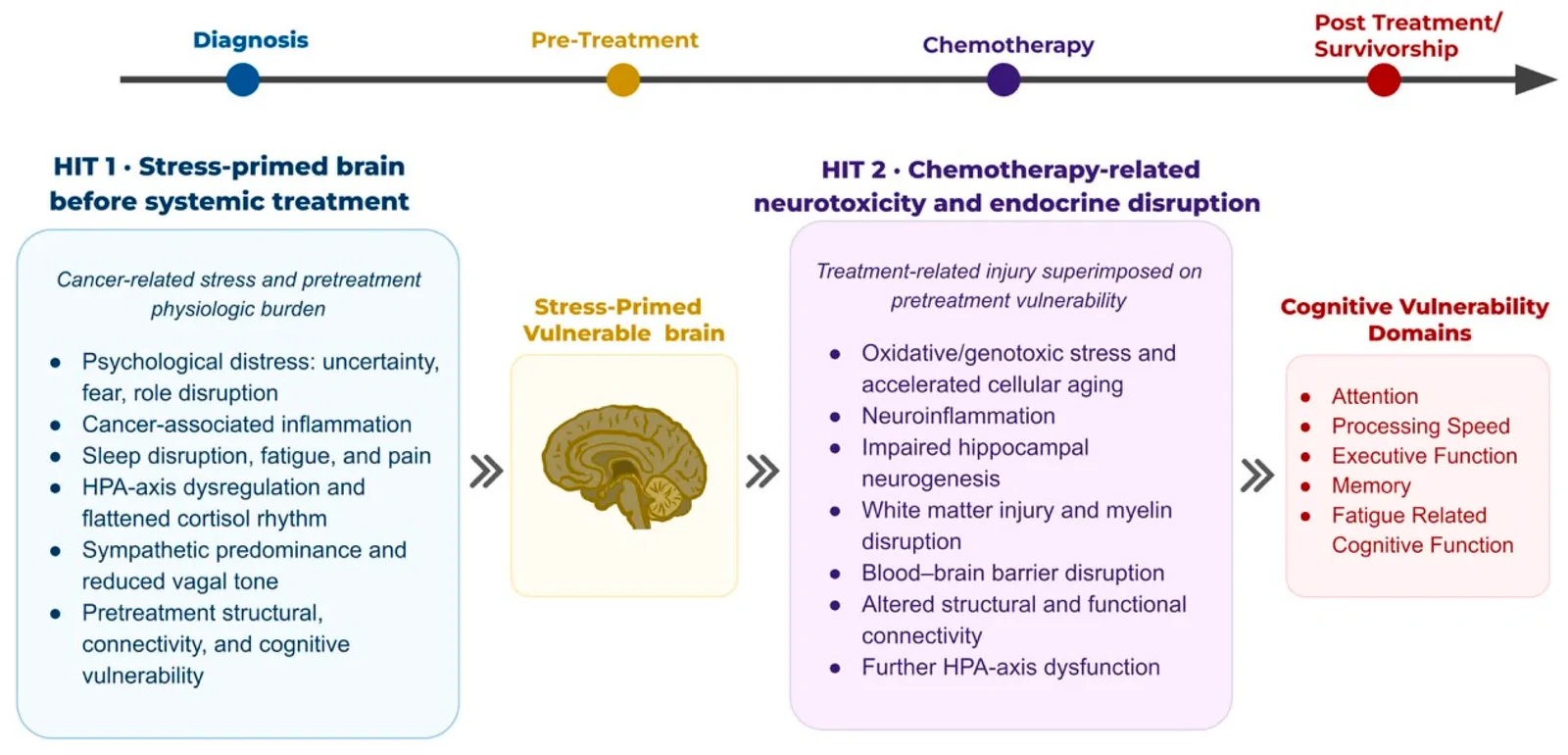
Proposed two-hit model of cognitive vulnerability in cancer. Arrows depict the proposed integration of these findings within the two-hit framework and should not be interpreted as establishing a causal sequence. Abbreviations: HPA, hypothalamic–pituitary–adrenal.

This review therefore adopts a two-hit framework. In the first hit, interacting cancer-, host-, and stress-related factors may create or amplify neural vulnerability before treatment. In the second, chemotherapy may add neurotoxic injury and further cognitive burden (Figure 1). This framework is intended as an organizing model rather than a proven causal sequence. The review focuses on adults with non-central nervous system cancers; pediatric populations and patients with primary brain tumors fall outside its scope.

## 2. Methodology

A literature search was performed using PubMed/MEDLINE and electronic resources accessed through the Midwestern University Library for peer-reviewed English-language articles published up to 25 August 2026. No lower publication-date restriction was applied. Search terms included combinations of keywords related to cancer-related cognitive impairment, chemotherapy-associated cognitive impairment, chronic and psychological stress, hypothalamic–pituitary–adrenal axis function, cortisol and glucocorticoid signaling, neuroinflammation, neuroimaging, autonomic function, and interventions relevant to cognitive vulnerability. Articles were selected based on their relevance to the topic, methodological quality, and contribution to mechanistic, clinical, or translational understanding. The review focused on adults with non-central nervous system cancers, and both clinical and preclinical studies were considered. Pediatric populations, primary central nervous system tumors, editorials, conference abstracts, and studies lacking sufficient methodological detail were excluded unless they provided highly relevant or seminal mechanistic or conceptual insights. Additional relevant articles were identified through manual screening of reference lists. Evidence was synthesized qualitatively to examine pretreatment cognitive vulnerability, chemotherapy-associated neurotoxicity, potential interactions with stress and HPA-axis dysregulation, and candidate interventions. Given the narrative nature of the review, no formal risk-of-bias assessment or quantitative synthesis was performed.

## 3. Epidemiology of Cancer, Psychological Stress, and Cognitive Burden

### 3.1. Global Cancer Incidence and Survivorship

The global cancer burden is large and continues to grow. In 2022, there were nearly 20 million new cases and 9.7 million deaths worldwide, and demographic pressures, particularly population growth and aging, are projected to increase annual incidence to approximately 35 million by 2050 [6]. Improvements in cancer detection and treatment have simultaneously increased the population living after a cancer diagnosis. Approximately 18.6 million cancer survivors were living in the United States as of 1 January 2025, a figure expected to surpass 22 million by 2035 [7]. As more patients survive longer, the cognitive, psychological, and functional consequences of cancer and its treatment have become increasingly important.

Cancer-related cognitive impairment (CRCI) can affect memory, attention, concentration, processing speed, and executive function. Its reported prevalence varies according to the population studied, timing of assessment, and method used to define impairment. A systematic review of longitudinal breast cancer studies found cognitive impairment in approximately 25% of patients before therapy, 24% after therapy, and 21% at follow-up extending to one year [1] (Table 1). A separate systematic review and meta-analysis found prevalence estimates of 21–34% using neuropsychological test batteries, compared with 44% by self-report and 16% using brief cognitive screening instruments [4]. These findings demonstrate that cognitive impairment can precede treatment and that prevalence estimates depend substantially on assessment methodology.

### 3.2. Psychological Stress in Cancer Populations

Psychological distress often emerges around the time of diagnosis, before systemic treatment begins. Patients may face uncertainty about prognosis, difficult treatment decisions, disruptions in work and family roles, financial strain, and fears related to pain, disability, recurrence, or death [3]. A 2024 meta-analysis of 84 studies estimated the overall prevalence of anxiety symptoms or disorders among patients with cancer at 23%, with a higher prevalence among women than men—31% compared with 20% [8]. Considerable heterogeneity accompanied these estimates because studies differed in cancer populations and methods of assessing anxiety.

Individual pretreatment cohorts may report substantially higher levels of psychological distress. Among patients with newly diagnosed locally advanced breast cancer assessed before neoadjuvant chemotherapy, anxiety was present in 47.85%, depression in 59.62%, and overall psychological distress in 65.07% [9]. These estimates are specific to this population and should not be generalized to all patients with cancer. Nevertheless, they demonstrate that considerable psychological burden may already be present before systemic treatment. Psychological symptoms may also persist into survivorship; a recent meta-analysis estimated anxiety in 24.4% and depression in 23.7% of cancer survivors [10].

The relationship between stress and cancer is complex and bidirectional. Cancer and its treatment can generate psychological and physiological stress, while chronic stress may influence inflammatory, neuroendocrine, autonomic, and immune pathways relevant to both systemic and neural function [3]. Current epidemiologic evidence, however, does not support describing psychological stress as an established independent cause of cancer. In the context of CRCI, the more relevant question is whether cancer-related psychological and physiological stress contributes to cognitive vulnerability before treatment.

### 3.3. Separating Cancer-Related from Chemotherapy-Related Brain Effects

Evidence of cognitive impairment before systemic therapy indicates that post-treatment deficits cannot be attributed solely to chemotherapy [1,2]. Potential contributors include psychological distress, cancer-associated physiological changes, fatigue, sleep disruption, pain, comorbid conditions, and other disease-related factors [2].

Studies enrolling patients only after chemotherapy has begun cannot determine how much cognitive difficulty was already present and how much developed or worsened during treatment. At the same time, post-treatment studies demonstrate cognitive changes and alterations in structural and functional brain networks associated with chemotherapy exposure [4,5]. Within the proposed two-hit framework, cancer-related stress and physiological disruption may therefore establish an initial state of neural vulnerability, after which chemotherapy adds a second neurotoxic exposure. This remains a working hypothesis rather than a demonstrated causal sequence. Prospective studies beginning at diagnosis and following patients before, during, and after treatment will be necessary to distinguish pretreatment vulnerability from treatment-related change.

## 4. Neurobiological Pathways Linking Chronic Stress to Brain Dysfunction

### 4.1. HPA Axis and Circadian Cortisol Dysregulation

The hypothalamic–pituitary–adrenal (HPA) axis provides the principal neuroendocrine route by which psychological and physical stress are translated into a systemic physiological response. A stressor stimulates hypothalamic corticotropin-releasing hormone (CRH), which drives pituitary adrenocorticotropic hormone (ACTH) release; arginine vasopressin (AVP) acts alongside CRH as a co-regulatory factor and potentiates this response, particularly during chronic or repeated stress adaptation. ACTH then stimulates cortisol secretion from the adrenal cortex. Under acute conditions, the sequence is adaptive, mobilizing energy, modulating immune activity, enhancing arousal, and supporting the behavioral response to challenge [11,12,13,14].

**Figure 2:**
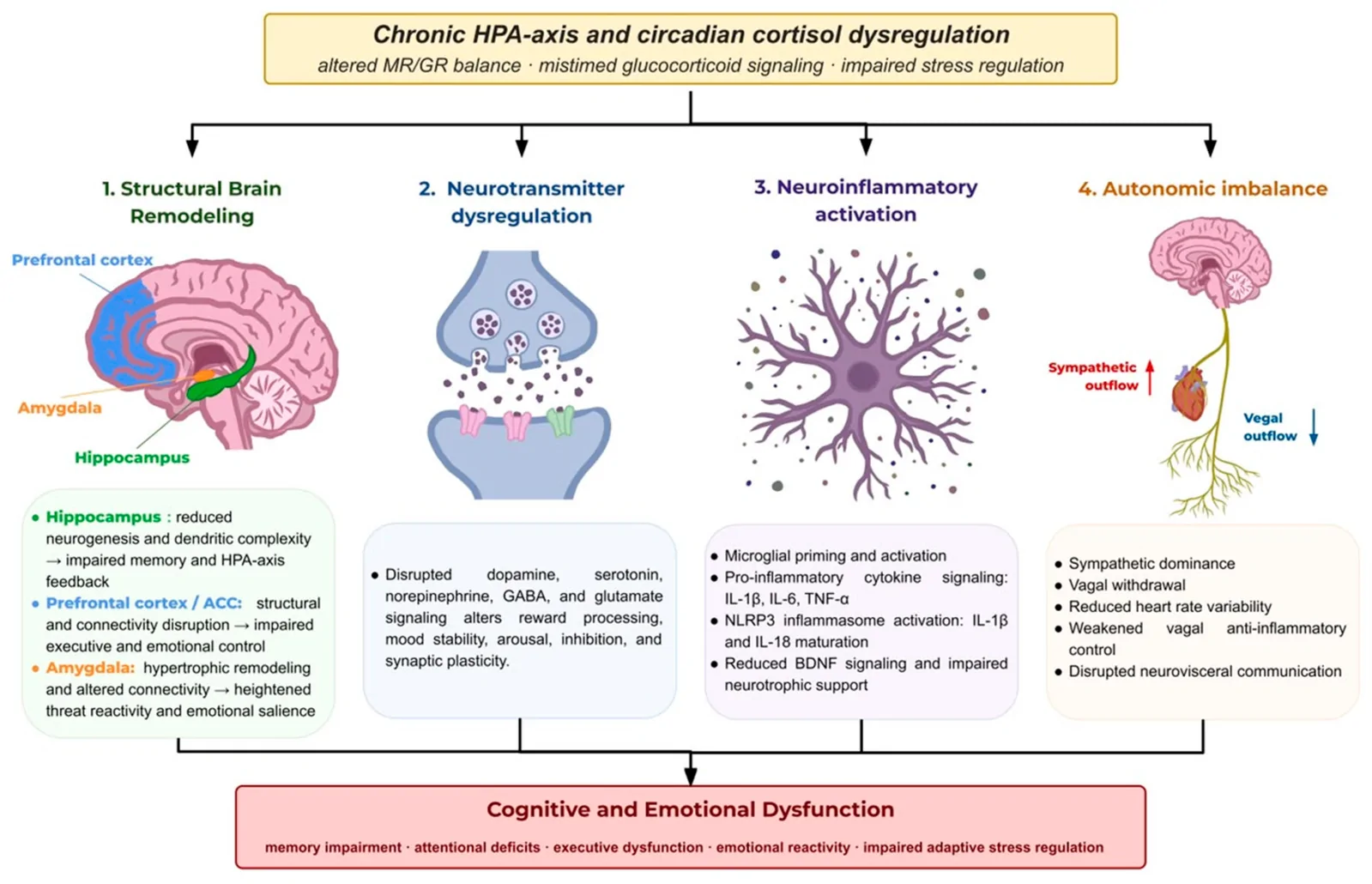
Integrated neurobiological pathways linking chronic stress to cognitive and emotional dysfunction. Evidence for these pathways derives from both preclinical and human studies, and their integrated role in pretreatment CRCI remains hypothesis-generating. Abbreviations: ACC, anterior cingulate cortex; BDNF, brain-derived neurotrophic factor; GABA, γ-aminobutyric acid; GR, glucocorticoid receptor; HPA, hypothalamic–pituitary–adrenal; IL-1β, interleukin-1β; IL-6, interleukin-6; IL-18, interleukin-18; MR, mineralocorticoid receptor; NLRP3, NLR family pyrin domain containing 3; TNF-α, tumor necrosis factor-α.

The problem emerges when glucocorticoid signaling no longer remains confined to that adaptive response. Cortisol acts throughout the brain through mineralocorticoid receptors (MRs) and glucocorticoid receptors (GRs), which differ in affinity, distribution, and function. MRs are preferentially occupied under basal conditions and support appraisal, emotional regulation, and cognitive stability, whereas GRs become increasingly engaged during stress and coordinate adaptation, energy mobilization, and negative-feedback termination of the stress response. Normal cognitive and emotional regulation therefore depends in part on the balance between the two systems. Repeated or prolonged stress can shift that balance toward maladaptive glucocorticoid signaling, weakening HPA-axis feedback regulation and increasing allostatic load [11,12,15,16] (Figure 2).

Cortisol exposure is defined not only by how much hormone is present but also by when it is present. Under physiological conditions, concentrations rise before awakening, peak shortly after waking, and decline progressively toward evening, with ultradian pulses superimposed across the day. This rhythmic organization coordinates glucocorticoid receptor activation and synchronizes metabolic, immune, autonomic, and neural processes with the sleep–wake cycle [11,12,16,17].

Chronic HPA-axis dysregulation disturbs that organization, often producing a blunted morning peak, a flatter diurnal cortisol slope, and mistimed glucocorticoid signaling. These changes should not be reduced to a simple question of whether cortisol is “high” or “low.” Absolute cortisol concentration, circadian rhythmicity, and stress responsiveness are distinct dimensions of HPA-axis function, and each may become dysregulated in a different way [11,17,18]. Disrupted rhythmicity has been associated with adverse physical and mental health outcomes, including immune and inflammatory dysfunction, fatigue, depression, cancer-related disease outcomes, and mortality [17]. HPA-axis dysregulation may therefore interact with several processes examined below, providing a plausible route through which prolonged psychological stress may alter brain structure, neurotransmitter signaling, neuroinflammatory activity, and autonomic balance.

### 4.2. Structural Brain Alterations Associated with Chronic Glucocorticoid Exposure

Chronic glucocorticoid exposure does not remodel the brain uniformly. Regions involved in cognition, emotional regulation, and HPA-axis feedback are particularly vulnerable because of their corticosteroid receptor density, reciprocal connectivity, and role in coordinating the adaptive stress response. The resulting pattern is therefore one of region-specific remodeling rather than generalized neurodegeneration. The hippocampus, prefrontal cortex, amygdala, and anterior cingulate cortex are among the most consistently affected structures, but they do not respond in the same direction [19,20,21].

The hippocampus is particularly susceptible because it supports memory and HPA-axis feedback and contains a high density of corticosteroid receptors [19,20,21]. Sustained cortisol exposure suppresses adult hippocampal neurogenesis, promotes dendritic retraction of cornu ammonis 3 (CA3) pyramidal neurons, reduces spine density, and is associated with hippocampal volume loss in animal models and human neuroimaging studies [22,23,24,25,26]. These changes impair memory and cognitive flexibility and may weaken hippocampal inhibition of the HPA axis [19,20].

The prefrontal cortex also shows stress-related retraction. Chronic stress or glucocorticoid exposure induces dendritic remodeling and spine loss in the medial prefrontal cortex, while human studies link chronic stress and cortisol exposure with reduced cortical thickness and gray-matter volume [26,27,28,29,30]. The anterior cingulate cortex shows related changes in gray matter, dendritic architecture, and functional connectivity [20,21,31,32]. These alterations are consistent with poorer executive function, attentional control, and emotional regulation [19,20].

The amygdala follows a different pattern. Chronic stress promotes dendritic arborization, spine formation, and increased synaptic connectivity in the basolateral amygdala [33,34,35], accompanied by greater anxiety, fear conditioning, and emotional reactivity [19,20]. Memory and control circuits may therefore retract as threat-responsive circuitry becomes more reactive. Stress also alters the neurotransmitter systems through which these regions communicate.

The anterior cingulate cortex adds another component to this stress-sensitive network. Together with interconnected dorsolateral prefrontal regions, the anterior cingulate cortex (ACC) contributes to cognitive control, conflict monitoring, emotional regulation, pain processing, and the integration of cognitive and affective information [19,20,21]. Chronic stress and stress-related psychopathology have been associated with changes in ACC gray matter, dendritic architecture, and functional connectivity [20,21,31,32]. These alterations track impaired emotional regulation, attentional control, and cognitive performance and may contribute to the executive dysfunction observed under prolonged glucocorticoid dysregulation [19,20].

The structural signature of chronic stress is therefore distributed but internally coherent: hippocampal and prefrontal systems supporting memory and cognitive control retract, amygdaloid circuits supporting threat responsiveness expand, and cingulate networks involved in integrating cognition and emotion are altered. These anatomical changes provide one substrate for chronic-stress dysfunction, but structure alone does not account for the resulting cognitive and behavioral phenotype. Stress also changes the chemical signaling through which these networks operate.

### 4.3. Neurotransmitter Dysregulation in Chronic Stress

Structural remodeling is accompanied by changes in neurotransmitter systems that regulate motivation, mood, attention, learning, and synaptic plasticity [20,36]. Chronic stress blunts dopaminergic reward and motivation circuits, contributing to anhedonia, reduced cognitive flexibility, and more habitual behavior [11,37], and can alter serotonergic tone through inflammation, impaired neurogenesis, and tryptophan shunting toward kynurenine. This contributes to the depressive symptoms, emotional dysregulation, and memory impairment associated with prolonged glucocorticoid exposure [20,21].

Noradrenergic signaling links cortisol more directly to arousal and emotionally salient memory. Norepinephrine regulates vigilance, attention, arousal, and stress-related memory, and cortisol interacts with noradrenergic signaling in amygdala- and hippocampus-dependent circuits during stress [11,38]. That interaction strengthens emotional salience and fear-related memory processing. When stress persists, the same relationship may contribute to hypervigilance, impaired attentional control, and heightened emotional reactivity [11].

The balance between γ-aminobutyric acid (GABA) and glutamate determines another level of vulnerability. GABA is the principal inhibitory neurotransmitter in the central nervous system, and chronic stress disrupts GABAergic signaling particularly within the medial prefrontal cortex, where remodeling of inhibitory interneurons and reductions in markers of GABAergic function have been observed [20,39]. The resulting loss of inhibitory regulation can disturb excitation–inhibition balance and increase vulnerability to anxiety, emotional dysregulation, and stress-related psychopathology [20,40].

Glutamate provides the excitatory counterpart. As the principal excitatory neurotransmitter of the central nervous system, it is essential for synaptic plasticity, learning, and memory. Chronic glucocorticoid exposure and repeated stress disrupt glutamate release, receptor trafficking, and synaptic plasticity in the hippocampus and prefrontal cortex [36,41,42,43]. These changes suppress long-term potentiation, facilitate long-term depression, and promote dendritic remodeling, impairing the synaptic adaptations required for learning and memory [36,44,45]. Glutamatergic dysfunction therefore represents one of the principal mechanisms connecting chronic stress to cognitive impairment and stress-related neuropsychiatric dysfunction [36].

Together, these changes convert the structural vulnerability described above into altered network function. Dopaminergic and serotonergic disruption affects motivation and mood, cortisol–norepinephrine interactions bias attention toward emotionally salient information, impaired GABAergic inhibition weakens regulatory control, and altered glutamatergic plasticity compromises learning and memory. Chronic stress therefore affects not only the architecture of stress-sensitive brain regions but also the chemical signaling required for those regions to function adaptively.

### 4.4. Neuroinflammation: Cytokines, Microglia, and the Stress-Brain Interface

The stress response extends beyond neurons. Chronic glucocorticoid exposure recruits neuroimmune pathways, placing microglia and inflammatory cytokine signaling alongside structural remodeling and neurotransmitter dysregulation as mechanisms of stress-related brain dysfunction [46,47].

Microglia bridge neuroendocrine disturbance and neuroinflammation. Prolonged exposure to stress hormones can shift these normally homeostatic immune cells toward a primed phenotype with altered morphology, immune function, and responsiveness to later inflammatory stimuli [46,47]. Activated microglia amplify inflammatory signaling through mediators including interleukin-1β (IL-1β), interleukin-6 (IL-6), and tumor necrosis factor-α (TNF-α), which can impair synaptic plasticity, reduce neurogenesis, alter neurotransmitter signaling, and disrupt neuronal communication in the hippocampus and prefrontal cortex [46,47,48].

The NLR family pyrin domain containing 3 (NLRP3) inflammasome supplies a more specific pathway. Its activation promotes caspase-1 activity and maturation of IL-1β and IL-18; in chronic stress models, this pathway increases local neuroinflammation, impairs hippocampal neurogenesis, and contributes to synaptic dysfunction and cognitive decline [48,49].

The relationship is bidirectional. Glucocorticoid dysregulation can promote microglial priming and inflammatory signaling, while inflammatory mediators can further disrupt HPA-axis regulation through limbic and hypothalamic circuits [46,47,48,49]. Reduced brain-derived neurotrophic factor (BDNF) signaling may add to this vulnerability by limiting neuronal survival, synaptic plasticity, and hippocampal neurogenesis [20,21,48]. The neuroinflammatory state associated with chronic stress is therefore not an isolated immune phenomenon. Sustained microglial activation, excessive cytokine signaling, and impaired neurotrophic support reinforce the same structural and synaptic vulnerabilities already produced by glucocorticoid dysregulation.

### 4.5. Autonomic Nervous System: Sympathetic Dominance and Parasympathetic Withdrawal

Chronic stress also alters brain–body regulation through the autonomic nervous system. Acute sympathetic activation supports vigilance and energy mobilization, while parasympathetic activity promotes recovery after the stressor resolves. Repeated stress shifts this balance toward sustained sympathetic drive and reduced vagal activity [50,51].

Sympathetic activation becomes maladaptive when adrenergic signaling persists beyond the acute threat. Sustained signaling maintains physiological hyperarousal and can promote inflammatory activity through effects on immune cells and peripheral tissues. In this setting, sympathetic overactivation becomes another chronic amplifier of neuroendocrine and neuroinflammatory dysregulation rather than a transient adaptive response [50,51].

At the same time, parasympathetic withdrawal removes an important counter-regulatory pathway. The vagus nerve is the principal parasympathetic conduit linking the brain to peripheral organs and plays a central role in limiting excessive inflammatory responses. Through the inflammatory reflex and its efferent cholinergic arm, vagal signaling suppresses production of pro-inflammatory cytokines including TNF-α, IL-1β, and IL-18 [52,53,54]. Reduced vagal tone therefore weakens an anti-inflammatory brake and allows inflammatory signaling to persist, further disrupting neuroendocrine and neural homeostasis [52].

Heart rate variability (HRV) provides a non-invasive readout of this autonomic balance. Defined as beat-to-beat variation in heart rate, HRV is strongly influenced by vagal activity [51,55]. Higher HRV generally reflects greater parasympathetic regulatory capacity and greater flexibility in responding to physiological and psychological demands [56]. Chronic stress, by contrast, is commonly associated with reduced HRV, diminished vagal modulation, increased autonomic rigidity, and impaired recovery from stress [51,57].

The same imbalance has consequences for brain function. Central autonomic networks spanning the prefrontal cortex, anterior cingulate cortex, insula, amygdala, hypothalamus, and brainstem integrate internal bodily signals, emotional salience, cognitive appraisal, and physiological responses to stress [56,57]. Persistent sympathetic activation and reduced vagal tone may therefore impair emotional regulation, pain processing, attentional control, and cognitive performance, linking peripheral autonomic imbalance to the cortical and limbic dysfunction described above [56,57].

## 5. Pre-Chemotherapy Brain Changes: Pretreatment Neural Vulnerability at Diagnosis

### 5.1. Structural and Connectivity Alterations Before Treatment Begins

The mechanisms described in Section 4 become clinically relevant only if corresponding brain abnormalities are detectable before chemotherapy begins. Neuroimaging evidence suggests that they can be. Even before cytotoxic treatment, patients with cancer may show structural and functional alterations across networks involved in cognition and emotional regulation. A comprehensive neuroimaging review linked the psychological stress and emotional burden surrounding a cancer diagnosis with functional and metabolic changes involving the prefrontal cortex, thalamus, cingulate cortex, hypothalamus, basal ganglia, and insula, regions also associated with anxiety, depression, posttraumatic stress symptoms, and psychological distress [58].

Resting-state imaging extends this evidence to limbic networks. D’Imperio et al. (2021) [59] examined women with breast cancer before chemotherapy using resting-state functional magnetic resonance imaging and identified altered connectivity involving the amygdala and hippocampus, structures central to emotion regulation and memory. Among patients with greater subjective memory complaints, resting-state connectivity was increased in the right amygdala and hippocampus and reduced in the left amygdala [58,59]. The finding places measurable neural alteration before the first cytotoxic exposure rather than exclusively after it.

### 5.2. Pre-Existing Cognitive Impairment Before Treatment

The pretreatment phenotype is not limited to neuroimaging. Treatment-naïve patients also demonstrate measurable cognitive abnormalities before receiving systemic therapy. Compared with healthy controls, treatment-naïve adolescent and young-adult cancer patients exhibited elevated baseline inflammatory markers, including IL-2, IL-4, IL-6, IL-8, IL-10, and IFN-γ, together with lower brain-derived neurotrophic factor (BDNF) levels [2]. These findings identify inflammatory and neurobiological disturbances present before treatment that potentially contribute to baseline cognitive vulnerability [60,61].

Pretreatment cognitive abnormalities vary by cancer population. Patients with Hodgkin lymphoma showed deficits in verbal learning and memory, processing/psychomotor speed, and abstraction/executive functions relative to matched controls [61], whereas pretreatment non-small-cell lung cancer (NSCLC) cohort found objective impairment in 36% of patients, with performance related to cognitive reserve and psychological factors [62]. Their presence before systemic treatment reinforces the distinction made in Section 3: cancer-related cognitive impairment cannot be treated as a phenomenon that begins only after chemotherapy.

That timing has practical consequences for interpretation. Without a cognitive baseline at diagnosis, disease-related cognitive changes and diagnosis-associated psychological distress can later be attributed to chemotherapy even when part of the impairment preceded treatment. Pretreatment assessment is therefore important for distinguishing subsequent treatment-associated change from cognitive abnormalities already present at diagnosis.

### 5.3. The Nocebo Effect: Anticipatory Cognition as a Potential Contributor

Pretreatment vulnerability can also be shaped by what patients expect treatment to do. The nocebo effect refers to adverse outcomes attributable to negative expectations and contextual or learning processes beyond the pharmacological effects of treatment. Its clinical consequences include greater symptom burden, poorer treatment adherence, and reduced therapeutic efficacy. Susceptibility reflects negative expectations, prior treatment experiences and conditioning, social observation, patient–clinician communication, contextual cues, negative mood and cognitive appraisal, personality characteristics, and the misattribution of pre-existing symptoms to treatment [63,64].

The effect is not confined to subjective symptom reporting. Experimental nocebo paradigms indicate that negative expectations can recruit physiological stress responses. In nocebo-hyperalgesia experiments, negative verbal suggestion has been associated with increased ACTH and cortisol, while Johansen et al. also observed greater cortisol elevation under nocebo conditions [65,66]. Cholecystokinin signaling has also been implicated in nocebo hyperalgesia, as cholecystokinin (CCK)-receptor antagonism with proglumide blocked experimentally induced nocebo-related pain amplification [67]. Anticipatory cognition can therefore recruit physiological stress pathways before or during treatment and add to the symptom burden surrounding chemotherapy exposure.

## 6. Chemotherapy-Associated Brain Changes: Evidence Across Cancer Populations

### 6.1. Mechanism and Structural Changes Associated with Chemotherapy Neurotoxicity

**Figure 3:**
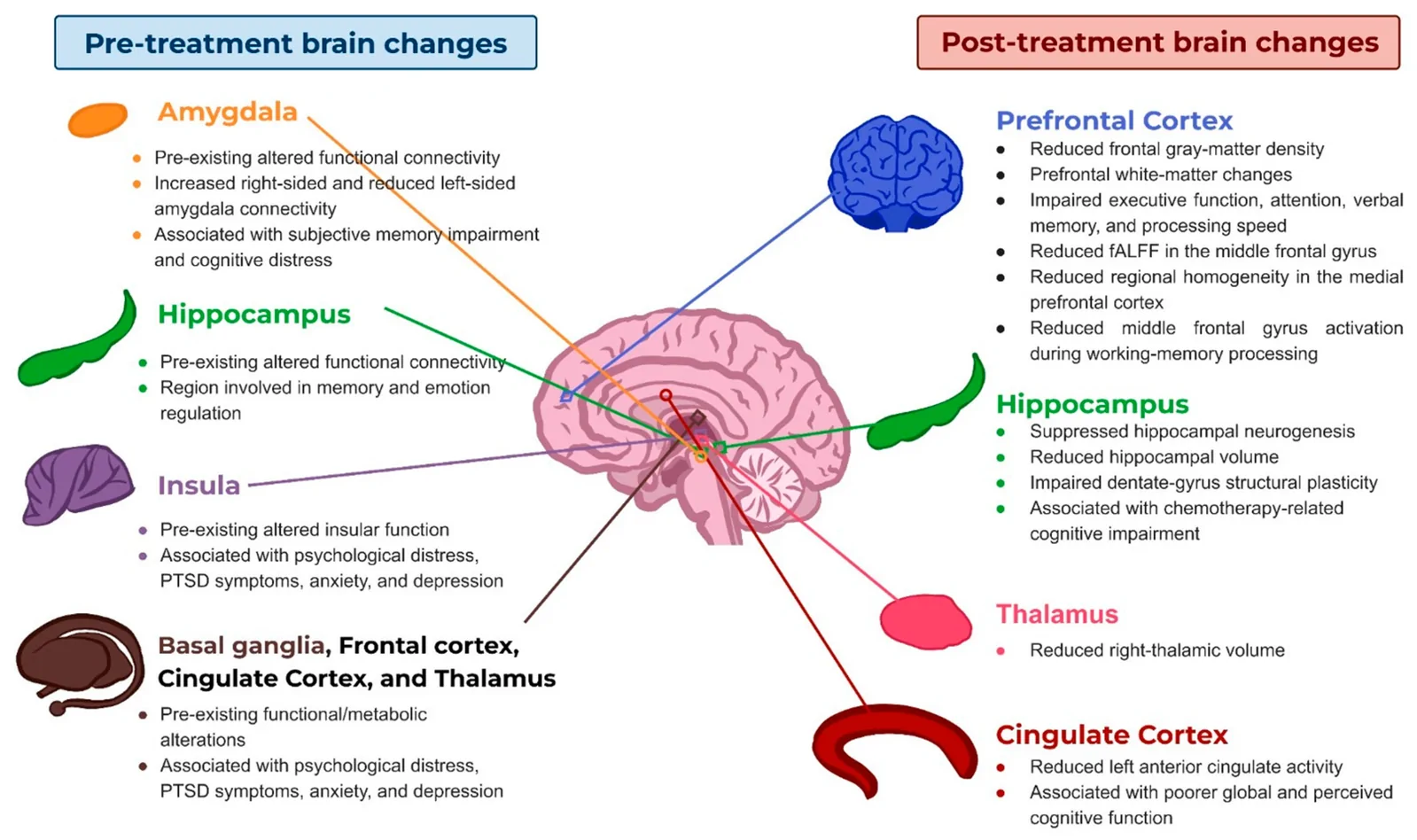
Pretreatment and post-treatment brain alterations associated with cancer and chemotherapy. The depicted abnormalities represent reported pretreatment and treatment-associated findings and do not independently establish their causal mechanisms. Abbreviations: fALFF, fractional amplitude of low-frequency fluctuations; PTSD, post-traumatic stress disorder.

If Section 4 and Section 5 define the biological and clinical basis of the first hit, chemotherapy provides a second source of injury through mechanisms that act directly on neural, glial, and vascular systems (Figure 3). One target is the proliferative cell population required for hippocampal plasticity. Chemotherapeutic agents can injure neural stem cells and markedly suppress adult hippocampal neurogenesis within the subgranular zone [68]. Loss of this dentate-gyrus plasticity has been implicated in chemotherapy-associated cognitive impairment [69], while neuroimaging studies in treatment-exposed patients demonstrate that breast-cancer survivors showed smaller total hippocampal volume and greater inward hippocampal deformation, particularly in the right hippocampus [68,69,70].

White matter represents a second point of vulnerability. Chemotherapy injures oligodendrocyte progenitor cells, the population responsible for maintaining and repairing myelin [69]. Chen et al. [71] identified longitudinal post-chemotherapy changes in white-matter integrity involving the corpus callosum and middle frontal gyrus, with neuropsychological performance associated with abnormalities in the corpus callosum, middle frontal gyrus, posterior limb of the internal capsule, and superior longitudinal fasciculus [69,71].

Chemotherapy can promote molecular features of accelerated aging through DNA damage and cellular senescence. Sanoff et al. [72] demonstrated sustained increases in the p16 inhibitor of cyclin-dependent kinase 4A (p16INK4a), alternative reading frame (ARF), and senescence-associated cytokines after chemotherapy but did not detect chemotherapy-associated telomere shortening. Broader reviews indicate that telomere attrition may occur with particular cancer treatments and treatment modalities, suggesting that the combination of persistent DNA lesions and telomeric degradation can arrest the cell cycle and drive vulnerable nervous-system components toward cellular senescence or programmed apoptosis, providing a substrate for longer-term cognitive and neurological decline [73].

The blood–brain barrier provides another route to the second hit. In an in vitro rat brain endothelial model, Branca et al. showed that oxaliplatin increased intracellular reactive oxygen species (ROS) and endoplasmic reticulum (ER) stress and altered the organization of zonula occludens-1 (ZO-1) and F-actin, producing a transient reduction in endothelial barrier tightness [74]. Once barrier integrity is weakened, inflammatory cytokines, systemic chemotherapeutics, and ROS can enter the central nervous system more readily, where they promote neuroinflammation, microglial and astrocyte activation, synaptic dysfunction, and neuronal cell death. These processes are associated clinically with deficits in executive function, processing speed, attention, working memory, and learning [73,74,75].

Glial injury extends beyond actively dividing progenitor populations. Non-mitotic cells such as astrocytes also develop altered cellular physiology after chemotherapy, contributing to a secondary neuroinflammatory environment and reducing the metabolic support available to surrounding neural tissue [69].

At the macroscopic level, these cellular processes are accompanied by measurable structural change. Longitudinal morphometric imaging demonstrated gray-matter reductions in bilateral frontal, temporal, and cerebellar regions and the right thalamus at an assessment performed approximately one month after chemotherapy completion [69]. The second hit is therefore visible across scales, from progenitor and glial injury to white-matter disruption, barrier compromise, and regional gray-matter loss.

### 6.2. Breast Cancer

Breast cancer provides some of the most developed clinical evidence for these treatment-associated brain changes. Prospective neuroimaging studies identify deterioration of white-matter microstructure after chemotherapy, including reduced fractional anisotropy and altered diffusion metrics within the corpus callosum, superior longitudinal fasciculus, inferior longitudinal fasciculus, corticospinal tract, and prefrontal white matter [76,77]. These abnormalities are associated with declines in attention, verbal memory, processing speed, and executive function, linking disruption of large-scale structural connectivity with impaired cognitive performance after treatment.

Gray-matter findings point in the same direction. Longitudinal MRI studies demonstrate reductions in frontal gray-matter density after chemotherapy accompanied by increased executive dysfunction [78]. Multimodal studies add evidence of increased serum neurofilament light chain, consistent with axonal injury, together with persistent abnormalities in white-matter organization and objective cognitive decline [76]. Across these modalities, the imaging and biomarker findings converge on structural injury involving both gray- and white-matter networks after chemotherapy [76,77,78,79].

The neuroendocrine system is altered alongside these structural effects. Cancer patients frequently exhibit a flattened diurnal cortisol trajectory rather than the normally steep decline in cortisol across the day, and this pattern has been associated with earlier mortality and persistent cancer-related fatigue [80,81]. Cortisol dysregulation has also been associated with impaired cognitive performance, suggesting that HPA-axis dysfunction may contribute to the cognitive burden observed during cancer treatment [80,81]. Morning salivary cortisol offers a non-invasive way to assess this system, but collection timing, adherence, sleep patterns, and day-to-day variability limit its interpretation as a standalone biomarker [81]. Its value is therefore greater when considered alongside behavioral, psychological, and neurobiological measures in identifying patients with stress-related vulnerability and in guiding approaches intended to mitigate cancer-related cognitive decline [80,81,82].

### 6.3. Lung Cancer

In lung cancer, pretreatment abnormalities may exceed post-treatment change. Simó et al. found more widespread white-matter damage and worse verbal memory in chemotherapy-naïve patients than in treated patients [83]. A later SCLC cohort found cognitive impairment in about one-third of patients at diagnosis and uncommon de novo impairment after chemotherapy [84]. Increased BBB leakage has also tracked poorer cognition in advanced disease [85]. Post-treatment imaging nevertheless shows focal gray- and white-matter abnormalities in paralimbic, frontal, and temporal regions [83]. The available evidence supports substantial disease-related vulnerability before chemotherapy, with additional treatment-associated change in some patients, while longitudinal evidence indicates that new chemotherapy-associated cognitive impairment is not universal.

### 6.4. Gastrointestinal Cancer

Functional imaging extends the second-hit evidence to colorectal cancer. Resting-state functional magnetic resonance imaging after systemic chemotherapy has identified altered intrinsic brain activity, particularly in anterior cingulate and frontal regions, together with poorer cognitive performance. Among patients treated with capecitabine- and oxaliplatin-based regimens, fractional amplitude of low-frequency fluctuation was reduced in the left anterior cingulate and middle frontal gyri. Anterior-cingulate fALFF was positively associated with global cognitive and perceived cognitive function, suggesting vulnerability of these regions to chemotherapy-related neurotoxicity [86].

### 6.5. Gynecological/Ovarian Cancer

Ovarian cancer adds converging structural and functional imaging evidence after platinum and taxane-based chemotherapy. Structural MRI in these patients showed reduced frontal and parietal gray-matter volume and reduced middle frontal and inferior parietal activation during N-back working memory, although neuropsychological performance did not differ from controls [87]. Resting-state imaging found reduced regional homogeneity in the middle frontal gyrus, medial prefrontal cortex, and superior parietal lobule [88]. Both studies were small, cross-sectional investigations from the same research group, so they do not provide independent replication.

### 6.6. Prostate Cancer

The prostate-specific literature on cognitive effects of cytotoxic chemotherapy is comparatively limited. A prospective multicenter cohort of older men with metastatic castration-resistant prostate cancer found that most patients maintained stable cognition or showed slight improvement; decline of at least 1.5 SD occurred in 0–6.5% depending on the measure, including 3 of 46 patients receiving chemotherapy [89]. Substantial deterioration was therefore not consistently observed, although the small prostate-specific evidence base limits broader conclusions [89].

### 6.7. Patients Receiving Adjuvant Treatment

Prospective studies of adjuvant chemotherapy are particularly informative because pretreatment assessment allows subsequent change to be measured against an established baseline. Adjuvant chemotherapy, given after surgery to eliminate residual malignant cells and reduce recurrence risk, is followed by measurable cognitive change in a subset of patients. Longitudinal studies identify declines after treatment in attention, learning, and processing speed, with some of these deficits persisting at longer-term follow-up [90].

Meta-analytic evidence is consistent with those findings. Across studies of adjuvant chemotherapy, treatment exposure was significantly associated with cognitive impairment, although the magnitude of impairment was generally small and varied by study design and cognitive domain [91]. The evidence therefore supports chemotherapy as a potential contributor to cognitive dysfunction while reinforcing the importance of pretreatment measurement of cognitive impairment when attributing subsequent changes to chemotherapy [90,91].

## 7. The Bi-Directional HPA Axis-Chemotherapy Relationship

The preceding sections treated stress-related brain injury and chemotherapy neurotoxicity as parallel exposures, but the evidence suggests potential interaction. Preclinical studies show that endogenous cortisol acting through the glucocorticoid receptor (GR) opposes the cytotoxic action of several chemotherapies at the same time as it damages stress-sensitive brain regions, while clinical studies indicate that cytotoxic treatment can perturb the HPA axis. These findings support a possible bidirectional relationship, although its contribution to CRCI in patients remains unproven.

### 7.1. Glucocorticoid Signaling and Chemotherapy Response

Preclinical studies indicate that glucocorticoid signaling can reduce the effects of microtubule-targeting chemotherapy. In the MIA PaCa-2 pancreatic line, paclitaxel-driven apoptosis is blunted by cortisol and restored by the selective GR modulator relacorilant; in OVCAR5 ovarian cells, relacorilant improved the efficacy of paclitaxel and the potency of platinum agents [92]. A combination screen in the same work found that microtubule-targeted agents consistently benefited from the addition of relacorilant, and the effect held in xenograft models, including MIA PaCa-2, HeLa, and a cholangiocarcinoma patient-derived xenograft, where tumor-cell apoptosis rose when relacorilant was added to paclitaxel. The pairing has since been tested clinically, where relacorilant with nab-paclitaxel produced durable disease control in taxane-resistant solid tumors [93].

Platinum agents subvert the same receptor by a different route. Rather than being restrained by circulating cortisol, cisplatin activates the GR directly: Pan et al. [94] reported that the drug binds cysteine-622 of the receptor and recruits it to the nucleus, where the activated receptor induces microtubule-associated serine/threonine kinase 1 (MAST1) and reactivates mitogen-activated protein kinase kinase (MEK) signaling to produce platinum resistance. Glucocorticoid exposure with dexamethasone reproduced the resistant phenotype, and MAST1 inhibition reversed it. Catecholamines released under sympathetic activation act together with glucocorticoids to support tumor-cell survival and blunt both chemotherapy and immunotherapy [95], so the stress hormones that accompany a cancer diagnosis converge on drug resistance from more than one receptor system.

### 7.2. The TSC22D3 Neuro-Immune Pathway

**Figure 4:**
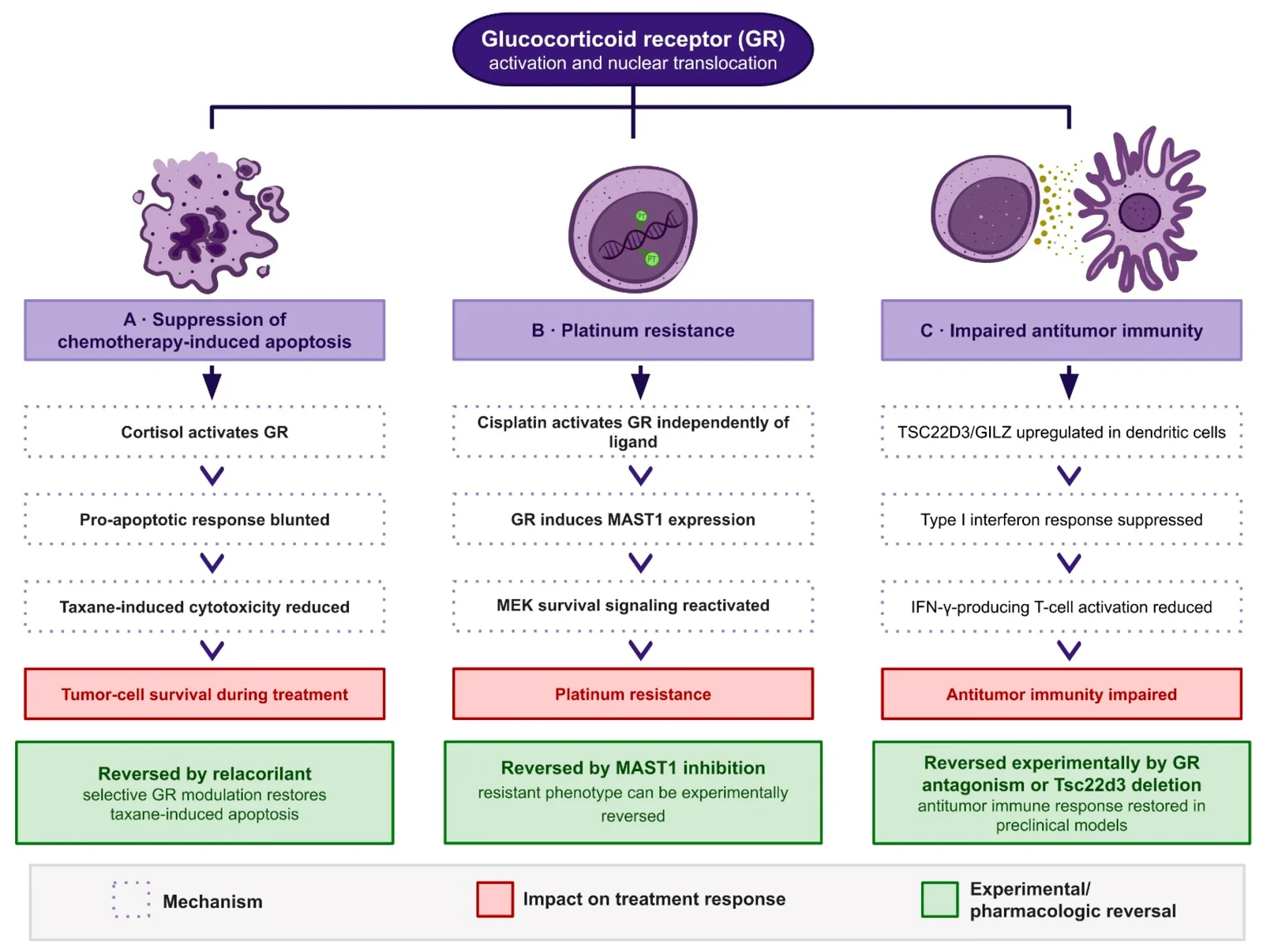
Glucocorticoid receptor signaling pathways that may impair cancer treatment response. These pathways are supported predominantly by preclinical and translational evidence; their contribution to CRCI in patients remains unestablished. Abbreviations: GILZ, glucocorticoid-induced leucine zipper; GR, glucocorticoid receptor; IFN-γ, interferon-γ; MAST1, microtubule-associated serine/threonine kinase 1; MEK, mitogen-activated protein kinase kinase; TSC22D3, TSC22 domain family member 3.

A more recent line of work connects psychological state to the immune arm of treatment response. In mouse models, Yang et al. [96] showed that chronic stress raised plasma corticosterone, which upregulated the glucocorticoid-inducible factor TSC22D3 (GILZ) in dendritic cells. TSC22D3 blocked type I interferon responses and the activation of interferon-γ-producing T cells, and enforced expression of the gene in dendritic cells was sufficient to abolish the therapeutic control of tumors. Administration of a glucocorticoid receptor antagonist (mifepristone), or dendritic-cell-specific deletion of Tsc22d3, reversed this effect; in patients, plasma cortisol, leukocyte TSC22D3 expression, and negative mood were closely correlated. The pathway supplies a defined molecular link from stress physiology to failure of antitumor immunity, and raises the possibility that lowering glucocorticoid tone could recover immunotherapy sensitivity, a mechanistic rather than purely palliative role for stress reduction (Figure 4).

### 7.3. Chemotherapy Directly Disrupts the HPA Axis

Chemotherapy can also alter HPA-axis function. In a systematic review of seventeen studies, Kanter et al. [97] found altered HPA function in sixteen; twelve reported increased baseline or hyperactive cortisol responses and four a decreased or blunted response, with fatigue, memory complaints, and disturbed sleep among the clinical correlates. Superimposed on this chronic picture is an acute pharmacological effect. Morrow et al. sampled serum cortisol hourly before and after treatment in twenty-three chemotherapy-naïve women with ovarian cancer and observed a significant fall in cortisol immediately after cisplatin or carboplatin infusion that did not occur on a separate day when the same procedures were performed without chemotherapy (*p* < 0.003) [98]. Because the drop appeared only in the presence of the cytotoxic drug, they attributed it to a direct effect rather than to circadian or psychological factors. Whether platinum suppresses adrenal steroidogenesis or pituitary ACTH output has not been resolved, so the mechanism of this acute perturbation remains open.

### 7.4. Perceived Stress and Cognitive Burden During Treatment

Patient-level evidence ties these mechanisms to the clinic. Ding et al. [82] recruited 450 patients receiving chemotherapy for breast cancer and tested four candidate routes from perceived stress to cognitive function within a single structural model: psychological resilience, social support, morning salivary cortisol, and the neutrophil-to-lymphocyte ratio. Perceived stress was inversely associated with cognition (direct effect −0.410, *p* < 0.001) [82]. Resilience and morning cortisol each carried a significant indirect effect, giving a total indirect effect of −0.231 (95% CI −0.364 to −0.102) [82], while paths through social support and the neutrophil-to-lymphocyte ratio did not hold and were dropped from the final model.

Higher perceived stress predicted lower morning cortisol (*p* = 0.0020), and lower morning cortisol predicted poorer cognition (*p* = 0.034) [82]. Blunting of the morning value, rather than the elevated output that a simple reading of stress physiology might anticipate, is the signature described in Section 4.1 as the leading edge of a flattened diurnal rhythm, so the finding converges with the argument that disrupted rhythmicity rather than absolute concentration is what carries consequences. The inference is limited by the cross-sectional design and by the small cortisol-mediated indirect effect compared with resilience (−0.023 vs. −0.208) [82]. Cortisol may contribute to the stress–cognition pathway, but its temporal position requires longitudinal sampling.

### 7.5. A Potential Bidirectional Feedback Loop

A flatter diurnal cortisol slope provides one link across the proposed cycle. In the meta-analysis by Adam et al. [17], flatter slopes were most strongly associated with immune and inflammatory burden (r = 0.288) and were also associated with cancer diagnosis and progression (r = 0.231) and mortality (hazard ratio 1.63 after removal of two extreme outliers). Chemotherapy can further perturb HPA function [98,99], while glucocorticoid signaling may suppress chemotherapy-induced apoptosis, promote platinum resistance, and impair antitumor immunity [92,94,96]. Patient-level data also place cortisol within the path from perceived stress to cognition, although its indirect effect was modest [82]. Together, these findings place cortisol at the intersection of neurocognitive and oncological processes, but do not establish a self-reinforcing causal cycle in patients. Circadian dysregulation is therefore best viewed as a candidate integrative marker rather than a demonstrated driver of CRCI.

## 8. Candidate Interventions Targeting Pretreatment Vulnerability

### 8.1. Rationale

If stress-related cortisol dysregulation precedes chemotherapy and compounds its neurotoxicity, then reducing that load becomes a candidate modifier of treatment response, not only a matter of comfort. Two features of the evidence reviewed above shape how such interventions should be designed. The stress-related circadian dysregulation framework [17] emphasizes restoring a normally steep diurnal decline rather than to lower total cortisol. The TSC22D3 pathway [96] provides a second rationale, as reducing glucocorticoid drive, whether behaviorally or pharmacologically, may recover a component of antitumor immunity that stress had suppressed. These approaches remain mechanistically motivated, but evidence that they prevent CRCI is currently limited.

### 8.2. Psychoeducation and Nocebo Mitigation

Because anticipatory expectation engages the stress-response pathways set out in Section 5.3, patient expectation is itself a modifiable point of intervention [63]. In a randomized trial of 100 patients beginning first-line chemotherapy for gastrointestinal cancer, those given a brief structured explanation of the nocebo effect reported lower adverse-event intensity at twelve weeks than an attention-matched control group (mean difference 4.04, 95% CI 0.72–7.36, Cohen’s d = 0.480) [64], an effect carried mainly by non-specific side effects. A review of forty-three reviews identified nine categories of nocebo risk factors, several of which, including information framing and patient–clinician communication, are open to modification during routine care [99]. Consent conversations and the wording used to prepare patients for treatment are therefore a low-cost lever on symptom burden.

### 8.3. Autonomic Restoration

Because chemotherapy may worsen the sympathetic dominance and vagal withdrawal described in Section 4.5, interventions that restore parasympathetic tone may address a specific mechanism rather than general wellbeing. Heart-rate-variability biofeedback, a breathing-based method for raising HRV as an index of emotional and cognitive self-regulation, was associated with improvements in sleep, pain, fatigue, depression, and anxiety across the studies gathered in a scoping review by Spada et al. [100]. Yoga has been tested directly against chemotherapy’s autonomic cost: in an eighteen-week randomized trial of 68 breast cancer patients receiving anthracycline-based treatment, groups were comparable at baseline, but after chemotherapy, the usual-care group showed higher resting heart rate and lower vagal indices, including the root mean square of successive differences (RMSSD), percentage of successive normal-to-normal intervals differing by more than 50 ms (pNN50), and high-frequency power, with a shift toward sympathetic predominance (higher low-frequency power and LF/HF ratio), while the yoga group was protected from this deterioration [101]. At the level of structured exercise, a meta-analysis of six trials in 272 patients and survivors found that training improved low-frequency power (SMD 0.97), high-frequency power (SMD 1.49), and the low-to-high-frequency ratio (mean difference −0.32) [102] relative to controls, with resistance and endurance formats most effective.

### 8.4. Psychological and Pharmacological Approaches

Cognitive behavioral therapy directed at anticipatory anxiety targets the same expectation circuitry that drives nocebo responses; by reducing the anxiety and neuroticism that Grosso et al. [99] identify as risk factors, reappraisal-based approaches should lower both distress and its autonomic and endocrine sequelae. On the pharmacological side, GR antagonism has a dual rationale: relacorilant restores chemotherapy-induced apoptosis and augments taxane efficacy [92,93], and, because it dampens glucocorticoid signaling, it may in principle reduce cortisol-driven strain on stress-sensitive neurons, though this neuroprotective extension is inferred from mechanism and has not been tested clinically. Beta-adrenergic blockade addresses the sympathetic arm: a week of preoperative propranolol reduced pro-metastatic gene expression in breast tumors [103], although the clinical benefit of beta-blockade appears to depend on tumor type and context and should not be generalized. Anti-inflammatory adjuncts follow from the finding that elevated cytokines and reduced BDNF accompany cognitive deficits before treatment begins [2] and from the strong association between a flat cortisol slope and immune burden [17], but they remain a mechanistic proposal pending trial evidence. Chronotherapy, timing cytotoxic delivery to circadian phase, reduced toxicity and raised the objective response rate for oxaliplatin-based regimens in metastatic colorectal cancer [104], though a later phase III trial found the survival advantage confined to men [105]; the approach is promising, but its efficacy data are mixed.

### 8.5. Sleep and Circadian Restoration

Sleep disruption both flattens the diurnal cortisol rhythm and worsens as that rhythm degrades, which makes sleep one of the more accessible routes to a steeper slope. In 77 cancer survivors with clinical fatigue, a four-week home light-therapy intervention re-steepened the diurnal cortisol slope (B = −0.02, Cohen’s d = 0.57) and raised total cortisol output [106]. The change occurred under both bright-white and dim-red light and did not statistically account for the improvement in fatigue, so light exposure is best regarded as a plausible means of circadian repair rather than a demonstrated mediator of symptom relief.

### 8.6. Clinical Integration

The through-line for practice is timing. Psychological state and cognition should be assessed at diagnosis rather than after treatment begins so that stress-related deficits present before the first infusion are not later misattributed to chemotherapy. Taken together, these measures point toward survivorship care organized across oncology, endocrinology, and psychology, in which the cortisol slope, autonomic tone, and cognitive trajectory are followed as related signals rather than in isolation.

## 9. Conclusions and Future Directions

Cancer-related cognitive impairment may begin before chemotherapy and persist through treatment. This pattern supports a two-hit framework in which multifactorial pretreatment vulnerability precedes additional chemotherapy-associated neurotoxicity [1,2,4,5]. However, evidence for the proposed sequence remains largely indirect and associative; much of the clinical literature is weighted toward breast cancer, and HPA-axis dysregulation should be viewed as one interacting pathway among tumor biology, inflammation, psychological distress, fatigue, sleep disruption, and pain.

Future research should use prospective, longitudinal designs beginning at diagnosis and continuing before, during, and after treatment. Establishing pretreatment cognitive and psychological baselines will be important for distinguishing pre-existing vulnerability from chemotherapy-related change. Studies integrating cognitive testing with measures of psychological distress, sleep, neuroendocrine function, inflammation, autonomic function, and neuroimaging may help clarify how these factors interact and identify patients at greatest risk. Recognizing that cognitive vulnerability may begin before treatment could ultimately support earlier assessment and more targeted strategies to preserve cognitive function throughout cancer care.

## Figures and Tables

**Table 1:** Reported Prevalence of Cancer-Related Cognitive Impairment and Psychological Distress Across the Cancer Trajectory.

Outcome	Population/Treatment Phase	Reported Prevalence	Source
Cancer-related cognitive impairment	Before therapy; breast cancer	25%	[1]
Cancer-related cognitive impairment	After therapy; breast cancer	24%	[1]
Cancer-related cognitive impairment	Follow up to 1 year; breast cancer	21%	[1]
Cancer-related cognitive impairment	During and after chemotherapy; breast cancer	21–34% by neuropsychological testing; 44% by self-report; 16% by brief screening	[4]
Anxiety symptoms or disorders	Overall cancer populations	23%	[8]
Anxiety symptoms or disorders	Women versus men with cancer	31% vs. 20%	[8]
Anxiety	Pretreatment locally advanced breast cancer cohort	47.85%	[9]
Depression	Pretreatment locally advanced breast cancer cohort	59.62%	[9]
Psychological stress	Pretreatment locally advanced breast cancer cohort	65.07%	[9]
Anxiety	Cancer survivors	24.4%	[10]
Depression	Cancer survivors	23.7%	[10]

*Note.* Reported estimates reflect the populations, treatment phases, follow-up periods, assessment methods, and outcome definitions used in the individual studies and therefore should not be interpreted as directly comparable across studies.

## Data Availability

No new data were created or analyzed in this study. Data sharing is not applicable to this article.

## Abbreviations

The following abbreviations are used in this manuscript:

ACC	Anterior cingulate cortex
ACTH	Adrenocorticotropic hormone
ARF	Alternative reading frame tumor suppressor protein
AVP	Arginine vasopressin
BBB	Blood–brain barrier
BDNF	Brain-derived neurotrophic factor
CA3	Cornu ammonis 3
CI	Confidence interval
CRCI	Cancer-related cognitive impairment
CRH	Corticotropin-releasing hormone
DNA	Deoxyribonucleic acid
ER	Endoplasmic reticulum
fALFF	Fractional amplitude of low-frequency fluctuations
fMRI	Functional magnetic resonance imaging
GABA	γ-Aminobutyric acid
GILZ	Glucocorticoid-induced leucine zipper
GR	Glucocorticoid receptor
HPA	Hypothalamic–pituitary–adrenal
HRV	Heart rate variability
IFN-γ	Interferon-γ
IL	Interleukin
IL-1β	Interleukin-1β
IL-2	Interleukin-2
IL-4	Interleukin-4
IL-6	Interleukin-6
IL-8	Interleukin-8
IL-10	Interleukin-10
IL-18	Interleukin-18
LF/HF	Low-frequency/high-frequency ratio
MAST1	Microtubule-associated serine/threonine kinase 1
MEK	Mitogen-activated protein kinase kinase
MR	Mineralocorticoid receptor
MRI	Magnetic resonance imaging
NIRS	Near-infrared spectroscopy
NLRP3	NLR family pyrin domain containing 3
NSCLC	Non-small-cell lung cancer
p16INK4a	p16 inhibitor of cyclin-dependent kinase 4A
pNN50	Percentage of successive normal-to-normal intervals differing by more than 50 ms
PTSD	Post-traumatic stress disorder
RMSSD	Root mean square of successive differences
ROS	Reactive oxygen species
SCLC	Small-cell lung cancer
SMD	Standardized mean difference
TMS	Transcranial magnetic stimulation
TMS–NIRS	Transcranial magnetic stimulation–near-infrared spectroscopy
TNF-α	Tumor necrosis factor-α
TSC22D3	TSC22 domain family member 3
ZO-1	Zonula occludens-1
